# Relationship Between Real-time TDM-guided Pharmacodynamic Target Attainment of Continuous Infusion Beta-lactam Monotherapy and Microbiologic Outcome in the Treatment of Critically Ill Children With Severe Documented Gram-negative Infections

**DOI:** 10.1097/INF.0000000000004054

**Published:** 2023-07-24

**Authors:** Milo Gatti, Caterina Campoli, Maria Elena Latrofa, Stefania Ramirez, Tommaso Sasso, Rita Mancini, Fabio Caramelli, Pierluigi Viale, Federico Pea

**Affiliations:** From the * Department of Medical and Surgical Sciences, Alma Mater Studiorum, University of Bologna, Bologna, Italy; † Clinical Pharmacology Unit, Department for Integrated Infectious Risk Management, IRCCS Azienda Ospedaliero-Universitaria di Bologna, Bologna, Italy; ‡ Infectious Diseases Unit, Department for Integrated Infectious Risk Management, IRCCS Azienda Ospedaliero-Universitaria di Bologna, Bologna, Italy; § Pediatric Intensive Care Unit, IRCCS Azienda Ospedaliero-Universitaria di Bologna, Bologna, Italy; ¶ LUM Metropolitan Laboratory, AUSL Bologna, Bologna, Italy.

**Keywords:** beta-lactams, continuous infusion, PK/PD target attainment, pediatric intensive care unit, microbiologic eradication

## Abstract

**Objectives:**

To explore the relationship between real-time therapeutic drug monitoring (TDM)-guided pharmacodynamic target attainment of continuous infusion (CI) beta-lactam monotherapy and microbiological outcome in the treatment of critically ill children with severe documented Gram-negative infections.

**Methods:**

Observational, monocentric, retrospective study of critically ill patients receiving CI piperacillin-tazobactam, ceftazidime, or meropenem in monotherapy for documented Gram-negative infections optimized by means of a real-time TDM-guided strategy. Average steady-state beta-lactam concentrations (C_ss_) were calculated for each patient, and the beta-lactam C_ss_/minimum inhibitory concentration (MIC) ratio was selected as a pharmacodynamic parameter of efficacy. The C_ss_/MIC ratio was defined as optimal if ≥4, quasi-optimal if between 1 and 4, and suboptimal if <1. The relationship between C_ss_/MIC and microbiological outcome was assessed.

**Results:**

Forty-six TDM assessments were carried out in 21 patients [median age 2 (interquartile range: 1–8) years]. C_ss_/MIC ratios were optimal in 76.2% of cases. Patients with optimal C_ss_/MIC ratios had both a significantly higher microbiological eradication rate (75.0% vs. 0.0%; *P* = 0.006) and lower resistance development rate (25.0% vs. 80.0%; *P* = 0.047) than those with quasi-optimal or suboptimal C_ss_/MIC ratios. Quasi-optimal/suboptimal C_ss_/MIC ratio occurred more frequently when patients had infections caused by pathogens with MIC values above the European Committee on Antimicrobial Susceptibility Testing clinical breakpoint (100.0% vs. 6.3%; *P* < 0.001).

**Conclusions:**

Real-time TDM-guided pharmacodynamic target attainment of CI beta-lactam monotherapy allowed to maximize treatment efficacy in most critically ill children with severe Gram-negative infections. Attaining early optimal C_ss_/MIC ratios of CI beta-lactams could be a key determinant associated with microbiologic eradication during the treatment of Gram-negative infections. Larger prospective studies are warranted for confirming our findings.

Sepsis is a leading cause of morbidity and mortality among critically ill children admitted to pediatric intensive care units (PICU), and the mortality rate in septic shock may be up to 50%.^1–3^ Gram-negatives are the predominant causative pathogens of healthcare-associated infections in the PICU,^4,5^ and the prevalence of multidrug-resistant isolates is growing.^4,5^

Beta-lactams are the backbone of treatment for severe Gram-negative infections. The recent Surviving Sepsis Campaign international guidelines for the management of septic shock in children recommended the need of optimizing antimicrobial dosing strategies according to well-established pharmacokinetic/pharmacodynamic (PK/PD) principles and specific drug properties for maximizing treatment efficacy.^6^ Beta-lactams are time-dependent agents and efficacy is related to the percentage of time of the dosing interval in which free plasma concentrations are maintained above the minimum inhibitory concentration (MIC) of the pathogen (%*f*T_>MIC_).^7^ However, choosing proper antibiotic dosing for granting appropriate exposure may be extremely challenging in critically ill children. Remarkable pathophysiologic alterations may affect the PK behavior of beta-lactams.^8–11^ Some real-world studies showed that the prevalence of underexposure and failure in attaining optimal PK/PD targets among critically ill children treated with a standard dose of beta-lactams may range from 65% to 90%.^12,13^ This may cause unfavorable clinical and microbiologic outcomes, prolonged organ dysfunction, and the development of antimicrobial resistance.^14,15^

Continuous infusion (CI) may represent the best strategy for maximizing beta-lactam PK/PD targets. Recent findings suggest that attaining a PK/PD target of 100%*f*T_>4-5 x MIC_ may minimize the emergence of antibiotic resistance during beta-lactam treatment among critically ill patients.^14,15^ Real-time therapeutic drug monitoring (TDM)-guided dosing adjustments of CI beta-lactams may represent a useful approach for optimizing promptly these targets among critically ill patients.^16,17^ Only a few studies assessed the impact that prolonged or CI may have on PK/PD target attainment and clinical outcome among critically ill children treated with beta-lactams.^18,19^ A cross-sectional survey on the implementation prolonged or CI of beta-lactams among neonatal sepsis patients found that only approximately 30% of respondents had access to this infusion modality.^18^ Likewise, a recent scoping review concerning beta-lactams TDM among septic children showed that administration by prolonged or CI was implemented only in 2 studies, even if none of these investigated the probability of attainment of aggressive PK/PD targets or the relationship with clinical outcome.^19^

The aim of this study was to explore the relationship between PK/PD target attainment and microbiologic eradication in critically ill children with severe documented Gram-negative infections treated with CI beta-lactam monotherapy.

## METHODS

### Study Design

Medical records of critically ill children admitted to the PICU of the Istituto di Ricovero e Cura a Carattere Scientifico Azienda Ospedaliero-Universitaria in Bologna from February 2021 to January 2022 who were treated with CI beta-lactams because of documented Gram-negative infections were retrospectively retrieved. Inclusion criteria were: age <18 years; targeted monotherapy with CI piperacillin-tazobactam, ceftazidime or meropenem because of documented Gram-negative infections; and TDM assessment within the first 72 hours. The study was conducted in accordance with the Declaration of Helsinki and was reviewed and approved by the Ethics Committee of Azienda Ospedaliero-Universitaria of Bologna (title: “Feasibility and utility of antimicrobial therapeutic drug monitoring in pediatric settings: a retrospective study”; No. 443/2021/Oss/AOUBo approved on 22nd June 2021). Signed informed consent was waived due to the retrospective and observational nature of the investigation according to hospital agreements.

### Beta-lactam Administration and Sampling Procedures

Piperacillin/tazobactam, ceftazidime and/or meropenem were prescribed at the discretion of the treating physician and/or of the infectious disease consultant in terms of therapeutic indication, dosage, and duration according to the current clinical practices implemented at the Istituto di Ricovero e Cura a Carattere Scientifico Azienda Ospedaliero-Universitaria in Bologna. Beta-lactam therapy was started with a loading dose (200 mg/kg for piperacillin-tazobactam, 50–100 mg/kg for ceftazidime and 20–40 mg/kg for meropenem over 2 hours), followed by a CI full maintenance dose (MD) of 400/50 mg/kg/day up to maximum 16/2 g/day for piperacillin/tazobactam; 150–200 mg/kg/day up to maximum 6g/day for ceftazidime; 60–120 mg/kg/day up to maximum 4g/day for meropenem. This strategy was routinely adopted in the early phase of sepsis/septic shock in all patients, including those with sepsis-associated acute kidney injury. The rationale was that of maximizing the likelihood of attaining early aggressive PK/PD targets while overcoming major pathophysiologic/iatrogenic factors that could have caused potential underexposure. Only in the presence of preexisting anuria/oliguria associated with severe renal dysfunction, maintenance doses were adjusted according to the revised bedside Schwartz formula for minimizing the risk of neurotoxicity. Fresh solutions were prepared every 24 hours for piperacillin-tazobactam and ceftazidime, and every 6–8 hours for meropenem due to its limited stability in aqueous solution at room temperature.^20^

Blood samples for assessing TDM steady-state concentrations (C_ss_) were collected first after 24–72 hours from the start of the treatment and subsequently whenever feasible. Total serum C_ss_ was measured by means of previously described methods^14^ at the Unique Metropolitan Laboratory of Bologna. Average C_ss_ was calculated in each single patient as the mean of all the C_ss_ values assessed (the first one before any dosage adjustment and the subsequent ones after eventual dosage adjustments). TDM results were made available within 6–8 hours via the intranet to the MD clinical pharmacologists, who provided expert interpretation [expert clinical pharmacologic advice (ECPA)] for personalizing beta-lactam exposure in each critically ill child.^16,17^ The TDM-guided ECPA was structured as an expert interpretation of the TDM result based on some specific factors. In regard to beta-lactams, dosing adaptation was defined by taking into account the in vitro susceptibility of the bacterial clinical isolate, the site of infection, and the pathophysiologic/iatrogenic features of each single patient [eg, body surface area, measured or estimated creatinine clearance (CL_Cr_), presence of sepsis/septic shock and/or of other co-morbidities, eventual application of renal replacement therapy], as previously reported.^16,17^

The MICs of the beta-lactams against the clinical isolates of Enterobacterales and Pseudomonas aeruginosa were determined by means of a semi-automated broth microdilution method (Microscan Beckman NMDRM1) and interpreted according to the European Committee on Antimicrobial Susceptibility Testing (EUCAST) clinical breakpoints.

The ratio between average C_ss_ and the MIC of the clinical isolate (C_ss_/MIC ratio) was selected as the PD determinant of beta-lactam efficacy in each single patient and defined as optimal when ranging from 4 to 8, quasi-optimal when ranging between 1 and 4, suboptimal if <1 and supra-optimal if >8. These thresholds were based on the findings of in vitro studies, experimental animal models and clinical studies showing that aggressive PK/PD targets based on C_ss_/MIC ratios and/or trough concentration/MIC ratios ≥4 (equivalent to 100% *f*T_> 4 × MIC_) may be associated with increased microbiologic eradication and suppression of resistance emergence to beta-lactams, as opposed to conservative PK/PD targets (40%–70% *f*T_> MIC_) commonly implemented in clinical trials.^14,15^ Dosage increases or decreases were applied whenever C_ss_/MIC ratios were <4 and >8, respectively.^16^

### Data Collection

Demographic (age, sex, weight, height, body surface area and underlying disease) and clinical/laboratory data [need for vasopressors, vasopressors dosage, requirement for mechanical ventilation, administration of loop diuretics, implementation of continuous renal replacement therapy (CRRT) during beta-lactam treatment, fluid and volume balance, 24-hour measured and estimated creatinine clearance, beta-lactam dosage, average C_ss_, overall number of ECPA, ECPA-recommended dosing adjustments, ECPA-recommended dosing adjustments at first TDM assessment, site/type of infection, Gram-negative isolates, MIC, microbiologic failure, resistance development and PICU mortality) were retrieved. Estimated CL_Cr_ (eCL_Cr_) was calculated according to the revised bedside Schwartz formula.^21^ Measured CL_Cr_ (mCL_Cr_) was defined according to 24-hour urine collection. Augmented renal clearance (ARC) was defined as a mCL_Cr_ value ≥130 mL/min/1.73 m^2^ in males and ≥120 mL/min/1.73 m^2^ in females coupled with a normal serum creatinine value.^22^ Fluid balance was defined as the difference between hydric input (ie, crystalloids, drug infusions and enteral/parenteral nutrition) and output (ie, diuresis, CRRT net removal and perspiration). Volume balance was defined as the difference between volume input (ie, colloids, albumin and blood components) and output (ie, drainages and ascites).

### Relationship Between PK/PD Target Attainment of CI Beta-lactam Monotherapy and Microbiologic Outcome

The relationship between the average PK/PD target attainment of CI beta-lactam monotherapy (in terms of optimal, quasi-optimal, suboptimal or supra-optimal C_ss_/MIC ratio) and the microbiologic outcome was assessed.

Microbiologic eradication was defined as the finding of negative cultures of samples collected at the infection site (namely bronchoalveolar lavage/bronchial aspirate, peritoneal fluid, urine or blood) in at least 2 subsequent assessments. Microbiologic failure was defined as the persistence of the same bacterial pathogen after ≥7 days from starting treatment in the follow-up cultures, as previously reported.^23^ Resistance development was defined as a MIC increase of the used beta-lactam against the clinical isolate beyond the EUCAST clinical breakpoint of susceptibility.

### Statistical Analysis

Descriptive statistics were used to describe the patient sample. Continuous data were presented as the median and interquartile range (IQR), and categorical variables were expressed as counts or percentages. Univariate analysis was assessed by means of the Mann-Whitney test in case of continuous variables and of the Fisher exact test or the χ^2^ test in case of categorical variables for comparing PICU patients with optimal and those with suboptimal/quasi-optimal beta-lactam PK/PD target attainment. The relationship between the average measured and estimated CLcr was assessed by means of simple linear regression. A *P* value of <0.05 was considered statistically significant. Statistical analysis was performed using MedCalc for Windows (MedCalc statistical software, version 19.6.1, MedCalc Software Ltd, Ostend, Belgium).

## RESULTS

Overall, 21 critically ill children were included in the study. Demographics and clinical features of the patients are reported in Tables 1 and 2. The median (IQR) age was 2 years (1–8 years), and 52.3% were female. The median (IQR) body surface area was 0.60 m^2^ (0.41–1.03 m^2^). The median (IQR) baseline 24-hour mCLcr and eCLcr were 62.5 mL/min (30.9–104.4 mL/min) and 139.6 mL/min/1.73m^2^ (91.8–171.5 mL/min/1.73m^2^), respectively (eCLcr vs. mCLcr, *r*^2^ = 0.28; see Figure, Supplemental Digital Content 1, http://links.lww.com/INF/F177). ARC based on mCLcr was found in 6 cases. Bowel obstruction or perforation (33.3%), pneumonia (23.8%), esophageal atresia (19.0%) and febrile neutropenia (14.3%) were the main causes for PICU admission. Sixteen of the 21 critically ill children needed loop diuretics, 10 needed mechanical ventilation and 6 needed vasopressors. CRRT was implemented in only 1 case. The median (IQR) pediatric index of mortality (PIM) at admission was 1.27 (0.39–6.51) and 1.04 (0.30–5.49) for PIM2 and PIM3, respectively. The overall PICU mortality rate was 4.8%.

**TABLE 1.** Demographics and Clinical Characteristics of Included Critically Ill Children

Patient Demographic	Patients (N = 21)
Age (years) [median (IQR)]	2 (1–8)
Gender (male/female) [n (%)]	10/11 (47.6/52.3)
Body weight (kg) [median (IQR)]	15 (8.1–28)
Body surface area (m^2^) [median (IQR)]	0.60 (0.41–1.03)
Measured CLcr (mL/min)* [median (IQR)]	62.5 (30.9–104.4)
Estimated CLcr (mL/min)* [median (IQR)]	139.6 (91.8–171.5)
Clinical variables	
Mechanical ventilation [n (%)]	10 (47.6)
Vasopressors [n (%)]	6 (28.6)
Continuous renal replacement therapy [n (%)]	1 (4.8)
Loop diuretics [n (%)]	16 (76.2)
Augmented renal clearance [n (%)]	6 (28.6)
PIM 2 score at admission [median (IQR)]	1.27 (0.39–6.51)
PIM 3 score at admission [median (IQR)]	1.04 (0.30–5.49)
Hydric balance (mL) [median (IQR)]	−83.8 (−225.4 to 32.5)
Volume balance (mL) [median (IQR)]	−8.8 (−14.3 to 14.6)
Underlying disease [n (%)]	
Bowel obstruction/perforation	7 (33.3)
Pneumonia	5 (23.8)
Esophageal atresia	4 (19.0)
Febrile neutropenia	3 (14.3)
Necrotizing enterocolitis	1 (4.8)
Drug-resistant epilepsy	1 (4.8)
Site of infection (targeted therapy)*	
Pneumonia	13 (61.9)
Bloodstream	7 (33.3)
Intrabdominal	4 (19.0)
Urinary tract	3 (14.3)
Beta-lactam treatment	
Piperacillin-tazobactam [n (%)]	10 (47.6)
Meropenem [n (%)]	10 (47.6)
Ceftazidime [n (%)]	1 (4.8)
Beta-lactam TDM	
Piperacillin/tazobactam average C_ss_ [median (IQR)]	44.8 (40.5–72.7)
Meropenem average C_ss_ [median (IQR)]	9.4 (6.9–17.7)
Ceftazidime average C_ss_ [median (IQR)]	20.2 (NA)
No. of TDM-guided ECPA per patient [median (IQR)]	2 (1–3)
C_ss_/MIC >4	16 (76.2)
C_ss_/MIC = 1–4	2 (9.5)
C_ss_/MIC <1	3 (14.3)
Expert clinical pharmacological advice	
Overall ECPAs	46
No. of dosages confirmed	31 (67.4)
No. of dosage increases	8 (17.4)
No. of dosage decreases	7 (15.2)
First TDM assessment within desired range	13 (62.0)
First TDM increase	4 (19.0)
First TDM decrease	4 (19.0)
Clinical outcome [n (%)]	
PICU mortality rate	1 (4.8)
Microbiological outcome [n (%)]	
Microbiological eradication	12 (57.1)
Resistance development	8 (38.1)

* Two patients with concomitant bloodstream infection + pneumonia; one patient with UTI + pneumonia; one patient with UTI + BSI; one patient with UTI + IAI + pneumonia.

CLcr indicates creatinine clearance; C_ss_, steady-state concentration; ECPA, expert clinical pharmacological advice; IQR, interquartile range; MIC, minimum inhibitory concentration; NA, not assessed; PICU, pediatric intensive care unit; PIM 2, pediatric index of mortality; PIM 3, pediatric index of mortality; TDM, therapeutic drug monitoring.

**TABLE 2.** Case-by-case Demographic and Clinical Features of 21 PICU Patients with Documented Gram-negative Infections

Id Cases	Age (years)/Sex	Height (cm) Weight (Kg)	Average Measured CLcr	Underlying Disease	Vasopressors	Mechanical Ventilation	CRRT	Loop Diuretics	Type of Infection	Pathogen	MIC (mg/L)	Beta-lactam dosing	C_ss_ av/MIC Ratio	Microbiological Eradication	Resistance Development
#1	6 months/F	56/4	1.4	Bowel obstruction	Dopamine 4 mcg/kg/min Dobutamine 4 mcg/kg/min	Yes	No	Yes	VAP	*E. cloacae*	8	PIT 600 mg/day CI	10.04	No	Yes (MIC > 16)
#2	1 month/M	40/1.6	1.8	Necrotizing enterocolitis	Dopamine 3 mcg/kg/min	No	No	No	IAI	*K. oxytoca*	8	PIT 528 mg/day CI	9.89	Yes	No
#3	1/F	80/15	42.8	Esophageal atresia	No	Yes	No	Yes	VAP	*P. aeruginosa*	8	MER 600 mg q8h CI	2.99	No	No
#4	1/F	65/7.6	30.3	Pneumonia	No	No	No	Yes	UTI HAP	*E. coliS. marcescens*	4 8	PIT 3 g/day CI	5.06	Yes (UTI) No (HAP)	No (UTI) Yes (HAP; MIC 16)
#5	1/F	65/8	34.5	Pneumonia	No	Yes	No	Yes	VAP	*S. marcescensE. coli*	0.12 0.12	MER 150 mg q8h CI	64.5	Yes	No
#6	1/F	68/5.4	102.0	Bowel obstruction	No	No	No	No	BSI	*Pantoea septica*	1	CTZ 1 g/day CI	20.1	Yes	No
#7	1/F	80/15	56.8	Esophageal atresia	No	No	No	Yes	BSI HAP	*P. aeruginosa*	8 8	MER 375 mg q6h CI	3.23	Yes (BSI) No (HAP)	No (BSI) Yes (HAP; MIC 32)
#8	1/F	73/8.1	31.1	Pneumonia	No	Yes	No	Yes	BSI VAP	*S. marcescens*	8 8	PIT 3 g/day CI	4.53	Yes (BSI) No (VAP)	No (BSI) Yes (VAP; MIC > 16)
#9	2/M	85/15.6	68.1	Pneumonia	No	Yes	No	Yes	VAP	*P. aeruginosa*	8	MER 250 mg q8h CI	0.56	No	Yes (MIC > 32)
#10	2/F	95/15	32.9	Pneumonia	No	Yes	No	Yes	IAI VAP UTI	*E. coli/KpP. aeruginosaC. freundii*	4 8 8 4	PIT 6.75 g/day CI	5.16	Yes	No
#11	2/M	87/12	131.3	Neoesophagous perforation	Dopamine 10 mcg/kg/min	Yes	No	Yes	VAP	*P. aeruginosa*	8	MER 250 mg q8h CI	0.83	No	Yes (MIC > 32)
#12	2/M	100/10	15.6	Esophageal - rectal atresia	No	No	No	Yes	HAP	*P. aeruginosa*	8	PIT 4.5 g/day CI	5.08	No	Yes (MIC > 16)
#13	3/M	88/14.5	132.1	Esophageal atresia	No	Yes	No	No	VAP	*P. aeruginosa*	8	MER 250 mg q8h CI	0.58	No	Yes (MIC > 32)
#14	4/F	109/19	4.5	Febrile neutropenia	Epinephrine 0.15 mcg/kg/min Dobutamine 8 mcg/kg/min Milrinone 0.5 mcg/kg/min	Yes	Yes	No	BSI	*E. coli*	0.12	MER 400 mg q6h CI	160.8	Yes	No
#15	5/M	98/17	78.2	Drug-resistant epilepsy	No	Yes	No	Yes	VAP	*E. cloacaeE. hormanechei*	8 4	PIT 10.8 g/day CI	4.74	Yes	No
#16	8/M	140/33	146.3	Bowel perforation	No	No	No	Yes	IAI	*E. coli*	8	PIT 4.5 g/day CI	6.08	Yes	No
#17	11/F	140/28	81.3	Bowel obstruction	No	No	No	No	HAP	*P. aeruginosa*	16	PIT 11.25g/day CI	4.78	Yes	No
#18	12/M	160/60	148.7	Bowel perforation	No	No	No	Yes	IAI	*E. coli*	8	PIT 18 g/day CI	15.37	Yes	No
#19	16/M	186/80	201.9	Febrile neutropenia	Dopamine 2 mcg/kg/min	No	No	Yes	BSI	*E. cloacae*	1	MER 1 g q6h CI	8.45	Yes	No
#20	17/F	150/51	123.9	Bowel obstruction	No	No	No	Yes	BSI UTI	*P. aeruginosa*	1 0.5	MER 500 mg q6h CI	12.9	Yes	No
#21	17/M	191/70.5	102.2	Febrile neutropenia	Norepinephrine 0.26 mcg/kg/min Epinephrine 0.04 mcg/kg/min	No	No	Yes	BSI	*E. coliKp*	0.12	MER 500 mg q6h CI	85.8	Yes	No

BSI indicates bloodstream infection; CI, continuous infusion; CLcr, creatinine clearance; CRRT, continuous renal replacement therapy; C_ss_ av, average steady-state concentration; CTZ, ceftazidime; F, female; HAP, hospital-acquired pneumonia; HSCT, hematopoietic stem cell transplant; IAI, intrabdominal infection; *Kp*, *Klebsiella pneumoniae*; M, male; MER, meropenem; MIC, minimum inhibitory concentration; PIT, piperacillin-tazobactam; UTI, urinary tract infection; VAP, ventilator-associated pneumonia.

Red color indicates microbiological failure.

The types of infection were pneumonia (13/21 cases, of which 9 were ventilator-associated pneumonia), bloodstream infections (7/21 cases), intrabdominal infections (4/21 cases) and complicated urinary tract infections (3/21 cases) (Table 1). Some patients had multi-site infections. Overall, 28 Gram-negative pathogens were isolated, being *Pseudomonas aeruginosa* (32.1%), *Escherichia coli* (25.0%) and *Enterobacter spp* (14.3%) the most frequent.

Piperacillin-tazobactam and meropenem were used in 10 patients each, and ceftazidime in 1 case. A total of 46 TDM-guided ECPA were performed, with a median (IQR) of 2 (1–3) per patient. The first TDM assessment was performed at 24, 48 and 72 hours in 2, 7 and 12 patients, respectively. At the first TDM assessment, beta-lactam dosing adjustments were needed in 8 of 21 cases (38.0%, of which 19.0% decreased and 19.0% increased). Specifically, in 4 patients, beta-lactam dosing was decreased (by 25%, 33% and 50% in 2 and 1 case each, respectively) according to the achievement of supra-optimal C_ss_/MIC ratio after the first TDM assessment. At subsequent TDM reassessments, beta-lactam dosing was confirmed in 3 patients, whereas a further dosing reduction by 25% was implemented in 1 case. In the other 4 patients, beta-lactam was increased (by 25%, 33% and 50% in 2, and 1 case each) according to the attainment of quasi-optimal (in 1 patient) or suboptimal (in 3 patients) C_ss_/MIC ratio. At subsequent TDM reassessments, further dosing increase by 33% was implemented in 1 patient with a suboptimal C_ss_/MIC ratio for attaining a quasi-optimal PK/PD target.

Overall, beta-lactam dosing adjustments were recommended in 15 of 46 ECPAs (32.6%, of which 17.4% increased and 15.2% decreased). The average C_ss_/MIC ratios were optimal in 16 cases (76.2%), quasi-optimal in 2 cases (9.5%) and suboptimal in 3 cases (14.3%).

**FIGURE 1.**
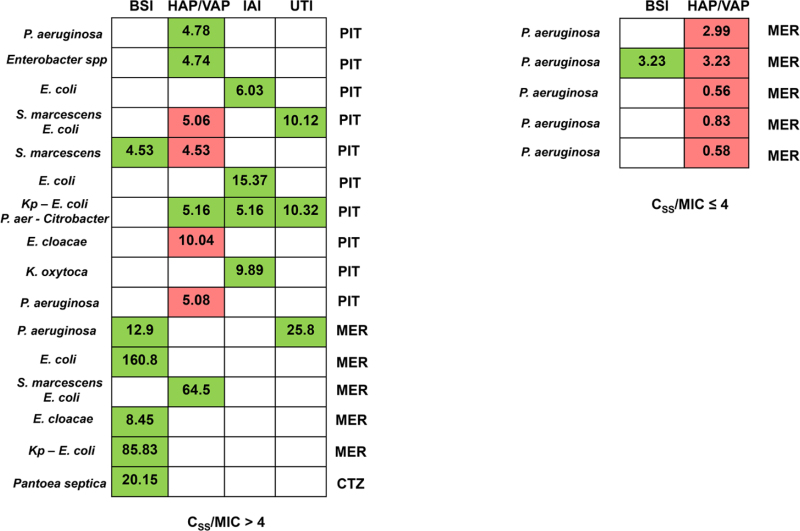
Relationship between pharmacokinetic/pharmacodynamic target attainment (expressed as average C_ss_/MIC ratio) and microbiologic outcome among critically ill children treated with CI beta-lactams. Green box, microbiologic eradication; red box, microbiologic failure; white box, absence of specific type of infection (in terms of infection site). Each row corresponds to a single patient. The C_ss_/MIC ratio is shown for each patient and defined as optimal if ≥4, or quasi-optimal/suboptimal if < 4. BSI indicates bloodstream infection; C_ss_, beta-lactam average steady-state concentrations; CTZ, ceftazidime; HAP, hospital-acquired pneumonia; IAI, intrabdominal infection; Kp, *Klebsiella pneumoniae*; MER, meropenem; MIC, minimum inhibitory concentration; PIT, piperacillin-tazobactam; UTI, complicated urinary tract infection.

A comparison between patients attaining optimal versus quasi-optimal/suboptimal C_ss_/MIC ratios of beta-lactams targeted therapy is reported in Table 3 and Fig. 1. Quasi-optimal/suboptimal C_ss_/MIC ratio occurred more frequently when patients had infections caused by pathogens with MIC values above the EUCAST clinical breakpoint (100.0% vs. 6.3%; *P* < 0.001). Beta-lactam dosage increases were needed more frequently in patients with quasi-optimal/suboptimal C_ss_/MIC ratio than in those with optimal C_ss_/MIC ratio (77.8% vs. 2.7%; *P* < 0.001).

**TABLE 3.** PICU Patients with Documented Infections: Comparison of Optimal vs. Quasi-optimal/Suboptimal PK/PD Target Attainment

Patient Variables	C_ss_/MIC ≥4 mg/L (n = 16)	C_ss_/MIC <4 mg/L (n = 5)	*P* Value
Age (years) [median (IQR)]	3 (1–11.3)	2 (1–2.25)	0.40
Gender (male/female) [n (%)]	7/9 (43.8/56.2)	3/2 (60.0/40.0)	0.64
Body weight (kg) [median (IQR)]	16 (7.6–42)	15.6 (15–16.2)	0.99
Body surface area (m^2^) [median (IQR)]	0.64 (0.38–1.30)	0.62 (0.59–0.64)	0.93
Measured CLcr (mL/min)* [median (IQR)]	34.5 (19.2–99.8)	68.1 (53.3–116.4)	0.35
Mechanical ventilation [n (%)]	6 (37.5)	4 (80.0)	0.15
Vasopressors [n (%)]	5 (31.3)	1 (20.0)	0.99
Continuous renal replacement therapy [n (%)]	1 (6.3)	0 (0.0)	0.99
Loop diuretics [n (%)]	12 (75.0)	4 (80.0)	0.99
Augmented renal clearance [n (%)]	4 (25.0)	2 (40.0)	0.60
PIM 2 score at admission [median (IQR)]	2.44 (0.77–6.56)	0.18 (0.13–5.46)	0.15
PIM 3 score at admission [median (IQR)]	1.40 (0.45–5.72)	0.07 (0.06–3.28)	0.17
Hydric balance [median (IQR)]	−61.5 (−413.4 to 27.1)	−106 (−180.7 to 71.5)	0.55
Volemic balance [median (IQR)]	−6 (−13.9 to 39)	−13.4 (−15 to −9.8)	0.26
Site of infection (targeted therapy)* [n (%)]			
Pneumonia	8 (50.0)	5 (100.0)	0.11
Bloodstream	6 (37.5)	1 (20.0)	0.62
Intrabdominal	4 (25.0)	0 (0.0)	0.53
Urinary tract	3 (18.8)	0 (0.0)	0.55
MIC of clinical isolates [n (%)]			
MIC below EUCAST clinical breakpoint	15 (93.7)	0 (0.0)	**<0.001**
MIC above EUCAST clinical breakpoint	1 (6.3)	5 (100.0)	
Clinical pharmacological advice [n (%)]			
No. of dosages confirmed	30 (81.1)	1 (11.1)	**<0.001**
No. of dosages increase	1 (2.7)	7 (77.8)	
No. of dosages decrease	6 (16.2)	1 (11.1)	
First TDM assessment within desired range	12 (75.0)	1 (20.0)	**<0.001**
First TDM increase	0 (0.0)	4 (80.0)	
First TDM decrease	4 (25.0)	0 (0.0)	
Microbiological outcome [n (%)]			
Microbiological eradication	12 (75.0)	0 (0.0)	**0.006**
Resistance development	4 (25.0)	4 (80.0)	**0.047**

* One patient with concomitant bloodstream infection + pneumonia; one patient with UTI + pneumonia; one patient with UTI + BSI; one patient with UTI + IAI + pneumonia in Css/MIC > 4 group; one patient with concomitant BSI + pneumonia in Css/MIC < 4 group.

CLcr indicates creatinine clearance; C_ss_, steady-state concentration; MIC, minimum inhibitory concentration; PICU, pediatric intensive care unit; PIM, Pediatric Index of Mortality; SD, standard deviation; TDM, therapeutic drug monitoring.

Microbiologic eradication was achieved in 12 of 21 cases. Overall, patients with optimal C_ss_/MIC ratios had both a higher microbiologic eradication rate (75.0% vs. 0.0%; *P* = 0.006) and a lower resistance development rate (25.0% vs. 80.0%; *P* = 0.047) than those with quasi-optimal or suboptimal C_ss_/MIC ratios.

Seventeen of 21 patients had early attainment of C_ss_/MIC ratio >4 (within the first 72 hours). Patients with early attainment of C_ss_/MIC ratio >4 showed a higher microbiologic eradication rate compared to those having early attainment of quasi-optimal or suboptimal C_ss_/MIC ratio (70.6% vs. 0.0%; *P* = 0.02).

Microbiologic failure occurred in 9 patients. All of these had pneumonia (due to *Pseudomonas aeruginosa* in 6 cases, *Serratia marcescens* in 2 cases and *Enterobacter cloacae* in 1 case; ventilator-associated pneumonia in 66.7% of cases). Antibiotic therapy was changed in 6/9 cases (combination with another anti-Gram-negative active agent in 4 cases and escalation to broader-spectrum antibiotic in 2 cases) and maintained unchanged in the other 3 cases (as significant clinical improvement was achieved anyway).

## DISCUSSION

Our study first assessed the relationship between PK/PD target attainment of CI beta-lactams and microbiologic outcome in the challenging scenario of PICU critically ill children with documented Gram-negative infections.

The findings suggest that administering beta-lactams by CI and optimizing PK/PD target attainment by means of a real-time TDM-guided ECPA approach could play a key role in ensuring microbiologic eradication. Some real-world studies found that intermittent and/or prolonged infusion of beta-lactams may allow optimal PK/PD target attainment only in a minority of critically ill children.^12,13^ Cies *et al*.^13^ found that standard doses of various beta-lactams (ampicillin, cefazolin, cefepime, cefotaxime, ceftaroline, doripenem, meropenem and piperacillin/tazobactam) by intermittent or by prolonged infusion over 3–4 hours failed in attaining a PK/PD target of 40% *f*T_> 4-6 × MIC_ in as much as 95% (78/82) of critically ill children with eCL_Cr_ >60 mL/min/1.73m^2^ and/or undergoing CRRT. Likewise, Van Der Heggen *et al*.^12^ recently reported that standard doses of amoxicillin-clavulanate, piperacillin-tazobactam or meropenem by intermittent infusion failed in attaining the aggressive PK/PD target of 100% *f*T_> 4 × MIC_ in 92.4% (145/157) of PICU patients, and that 2 independent predictors of subtherapeutic beta-lactam exposure were high glomerular filtration rate estimates and no need for vasopressors.

In this scenario, administration by CI may be a valuable strategy for maximizing the time-dependent activity of beta-lactams in critically ill pediatric patients. CI may allow the attainment of very aggressive PK/PD targets of beta-lactams, as previously reported in critically ill adults,^24,25^ often with lower doses than needed by intermittent infusion. Additionally, CI administration may be helpful in counteracting the neurotoxicity risk associated with the high peak levels achieved during intermittent infusion.^26,27^ The latter could be especially remarkable in neonates and toddlers, who could be at higher toxicity risk due to the immature status of the blood-brain barrier.^28,29^

The findings may support the role that a real-time TDM-based approach may have in improving outcomes among PICU patients treated with CI beta-lactams for severe documented Gram-negative infections. This strategy may allow the identification of patients not attaining optimal PK/PD targets of beta-lactams in the first 72 hours who may benefit from prompt dosing adaptation, thus minimizing, on the one hand, the risk of microbiologic failure and, on the other hand, that of drug-related toxicity. It is noteworthy that this approach grants the attainment of optimal PK/PD targets in most cases, often even when dealing with infections caused by pathogens with borderline susceptibility to beta-lactams. Additionally, it granted microbiologic eradication and prevention of resistance development in more than half of cases. Noteworthy, PICU patients with optimal PK/PD target attainment achieved microbiologic eradication more frequently compared to those with quasi-optimal or suboptimal ones. This is consistent with what was previously observed with both traditional and novel beta-lactams in critically ill adult patients.^14,15,30–34^

The need for a TDM-based approach is also supported by the fact that PICU patients may frequently have major pathophysiologic alterations that may affect the PK behavior of beta-lactams.^7^ Occurrence of ARC or transient acute kidney injury, vasopressors requirement, use of loop diuretics and negative fluid balance may be key determinants in affecting beta-lactam exposure.^8–11,35^ In this regard, it is worth mentioning that estimating glomerular filtration rate by means of the revised bedside Schwartz formula turned out to be unreliable in our cohort, as witnessed by the very poor correlation with the mCLcr. If renal function were only estimated, the glomerular filtration rate would have been overestimated by the revised bedside Schwartz formula, and most of our PICU patients would have been wrongly considered as having ARC. The unfortunate consequence of this could have been the inappropriate selection of too high doses of beta-lactams. Therefore, measuring CLcr must be considered the only effective and safe way for properly assessing renal function in PICU patients, as just previously shown.^36,37^

Microbiologic failures occurred only among PICU patients with pneumonia. The high failure rate in pneumonia could have been related to the high inoculum effect, which may have attenuated the effectiveness of beta-lactams,^38^ and/or to the limited penetration rate of beta-lactams into the epithelial lining fluid.^39,40^ Indeed, both of these could have been responsible for suboptimal PK/PD target attainment at the infection site, especially in those patients who had borderline optimal PK/PD targets, namely a C_ss_/MIC ratio of 4–5. Overall, this may support the contention that when treating pneumonia with CI beta-lactams probably more aggressive C_ss_/MIC ratios of 6–8 rather than of 4–8 should be considered for attaining microbiologic eradication, as recently suggested.^16^

We recognize that our study has some limits. The study assessed only a small cohort of critical pediatric patients and therefore should be considered simply as a proof-of-concept whose findings should be confirmed in larger prospective studies. The retrospective monocentric design should be acknowledged. The PK/PD analysis was based on total drug concentrations, even if no relevant impact on interpretation would be expected given the low plasma protein binding of the tested beta-lactams (ranging from <10% for meropenem and ceftazidime and approximately 20% for piperacillin). Average C_ss_ values were used for calculating C_ss_/MIC ratios in each single patient, whereas weighted approaches were not implemented. However, we believe that the detailed analysis of the relationship between PK/PD target attainment and microbiologic outcome in PICU patients with documented Gram-negative infections may represent a major strength.

In conclusion, administering beta-lactams by CI and personalizing treatment by means of a TDM-guided ECPA strategy may allow to maximize PK/PD target attainment in PICU critically ill children, and could be a key determinant for achieving microbiologic eradication when treating documented severe Gram-negative infections. Larger prospective studies are warranted for confirming our findings.

## Supplementary Material


