# Supplementary material for: Relationship Between Real-time TDM-guided Pharmacodynamic Target Attainment of Continuous Infusion Beta-lactam Monotherapy and Microbiologic Outcome in the Treatment of Critically Ill Children With Severe Documented Gram-negative Infections
Source: Pediatr Infect Dis J. 2023 Jul 24;42(11):975–82. doi: 10.1097/INF.0000000000004054 (PMC10569676; doi:10.1097/INF.0000000000004054)

**Supplemental Digital Content 1.** Correlation between average measured creatinine clearance (x-axis) and average estimated creatinine clearance (by means of the bedside revised Schwartz formula; y-axis). The relationship was poor (*R*^2^=0.28).


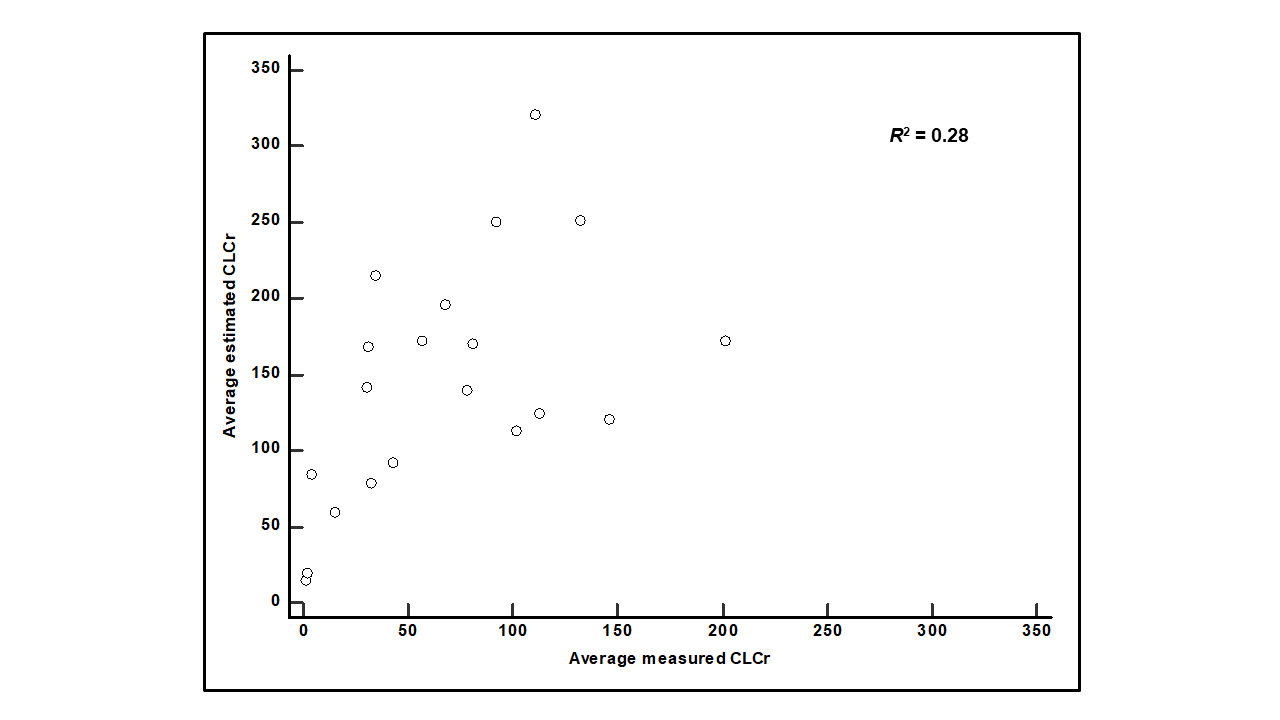

Supplement: Supplementary file 1 [file inf-42-0975-s001.docx]
